# Epidemiology and impact on all-cause mortality of sepsis in Norwegian hospitals: A national retrospective study

**DOI:** 10.1371/journal.pone.0187990

**Published:** 2017-11-17

**Authors:** Siri Tandberg Knoop, Steinar Skrede, Nina Langeland, Hans Kristian Flaatten

**Affiliations:** 1 Department of Medicine, Haukeland University Hospital, Bergen, Norway; 2 Department of Clinical Science, University of Bergen, Bergen, Norway; 3 Department of Anaesthesia and Intensive Care, Haukeland University Hospital, Bergen, Norway; 4 Department of Clinical Medicine, University of Bergen, Bergen, Norway; University of Florida, UNITED STATES

## Abstract

**Background:**

Although sepsis is the leading cause of death from infection, there are few population-level epidemiological sepsis reports. The impact of sepsis-related deaths on all-cause hospital mortality is insufficiently described, in particular in Europe where data are non-existent. The objective of this study was to provide nationwide epidemiological results on sepsis hospitalizations in Norway and to estimate sepsis’ contribution to overall hospital mortality in a European setting.

**Methods:**

We performed a retrospective study using data from the Norwegian Patient Registry and Statistics Norway. The occurrence, patient characteristics and outcomes of sepsis hospitalizations during the years 2011 and 2012 were estimated and compared with Norwegian population data. Sepsis was defined as organ dysfunction caused by a dysregulated host response to infection and identified with International Classification of Diseases 10^th^ revision codes.

**Results:**

We identified 18 460 sepsis admissions occurring in 13 582 individuals. The annual population incidence of hospitalized sepsis was 140 patients per 100 000 inhabitants; ranging from 10 to 2270 per 100 000 in different age groups and with statistically significant male predominance in all adult cohorts. Hospital mortality for sepsis admissions was 19.4% and overall, 26.4% of the included patients died while hospitalized for sepsis. Sepsis related deaths constituted 12.9% of all hospital fatalities, while hospitalizations with sepsis accounted for 1.0% of the total number of admissions and 3.5% of the total admission days during 2011 and 2012.

**Conclusions:**

This study confirms that hospitalized sepsis is frequent in Norway and a major contributor to hospital fatalities in a European setting. The incidence is higher among men than women. Sepsis is in particular a disease of the elderly, and its impact on health-care will assumingly continue to increase in parallel with an aging population. Improvements in treatment and survival of sepsis could influence population mortality, and sepsis should receive greater attention in official death statistics in the future.

## Introduction

Sepsis is the leading cause of death from infection and a major public health concern in most countries. Still, the epidemiology of this condition is insufficiently described. Population-level results on the incidence of hospital-treated sepsis exist for only eight countries around the world, including Norway as one of four European sites [1, 2]. The currently available Norwegian study is however from the year 1999, and thus of uncertain validity as the occurrence and outcome of sepsis has changed during the last decades [1, 3]. Hence, this study was conducted to gain updated results on the epidemiology of sepsis hospitalizations in Norway. Furthermore, a secondary objective was to investigate sepsis’ contribution to hospital fatalities, which previously has been surveyed in the United States (U.S.) only [4]. Since we were able to extract information from all Norwegian hospitals, we present the first estimate of sepsis’ impact on overall hospital mortality from complete nationwide data.

## Materials and methods

**Fig 1:**
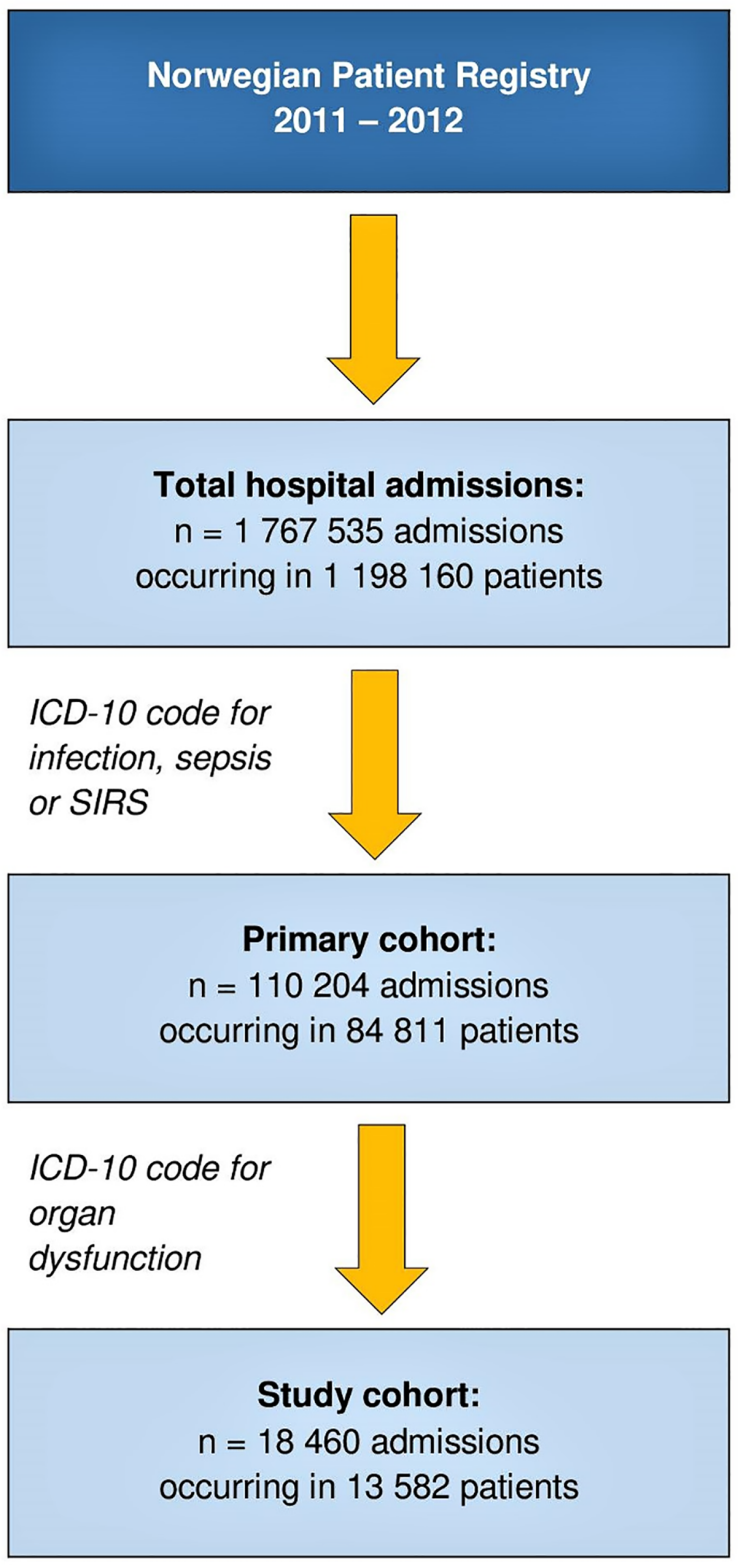
Diagram of the inclusion process.

This was a retrospective study combining hospitalization data from the Norwegian Patient Registry (NPR) and population data from Statistics Norway [5, 6]. The years 2011 and 2012 were chosen because these were the most recent years from which complete data were available when the study was conceived. The NPR is a national database run by the Norwegian Directorate of Health, containing information about all hospital admissions in Norway (patient data, dates of hospitalization, type of hospital and department, vital status at discharge and International Classification of Diseases 10^th^ revision (ICD-10) discharge codes). Reporting to the NPR is mandatory. In the current study, a primary search throughout the years 2011 and 2012 was performed by use of selected ICD-10 discharge codes for infections, systemic inflammatory response syndrome (SIRS), sepsis by causative microbes, and septic shock (Table 1). In this primary cohort, we then searched for the presence of up to eight additional ICD-10 discharge codes indicating acute organ dysfunction. Sepsis was defined as life-threatening organ dysfunction caused by a dysregulated host response to infection, inspired by the Third International Consensus Definitions for Sepsis and Septic Shock [7]. Accordingly, the final study cohort consisted of cases fulfilling one or several infection or sepsis related ICD-10 codes as well as one or several codes for acute organ dysfunction (Fig 1).

**Table 1:** ICD-10 codes used in this study.

ICD-10 code ^a^	Diagnosis
**Infection, sepsis or SIRS**	
A02.1	Salmonella sepsis
A20.7	Septicaemic plague
A21.7	Sepsis (generalized) tularemic
A22.7	Anthrax sepsis
A24.1	Acute and fulminating melioidosis
A26.7	Erysipelothrix sepsis
A32.7	Listerial sepsis
A39.2	Acute meningococcaemia
A40 (.0, 1, 2, 3, 8, 9)	Streptococcal sepsis
A41 (.0, 1, 2, 3, 4, 5, 8, 9)	Other sepsis
A42.7	Actinomycotic sepsis
A46	Erysipelas
A48.3	Toxic shock syndrome
A54.8	Other gonococcal infections
B37.7	Candidal sepsis
J09	Influenza due to identified zoonotic or pandemic influenza virus
J10	Influenza due to identified seasonal influenza virus
J13	Pneumonia due to Streptococcus pneumoniae
J14	Pneumonia due to Haemophilus influenzae
J15	Bacterial pneumonia, not elsewhere classified
J18 (.0, 1, 2, 8, 9)	Pneumonia, unspecified microbiology
J36	Peritonsillar abscess
J39	Other diseases of upper respiratory tract
J85	Abscess of lung and mediastinum
J86	Pyothorax
K65	Peritonitis
K81	Cholecystitis
M72.6	Necrotizing fasciitis
N10	Acute tubulo-interstitial nephritis
O85	Puerperal sepsis
P36	Bacterial sepsis of newborn
R57.2	Septic shock
R65 (.0, 1, 9)	Systemic Inflammatory Response Syndrome [SIRS] of infectious origin without (.0) or with organ dysfunction (.1), or not further specified (.9)
T81.4	Infection following a procedure
**Organ dysfunctions**	
R57	Shock
I50.9	Heart failure, unspecified
J80	Adult respiratory distress syndrome
J95	Postprocedural respiratory disorders
J96.0	Acute respiratory failure
N17	Acute renal failure
N99.0	Postprocedural renal failure
D65	Disseminated intravascular coagulation [defibrination syndrome]
D69	Purpura and other haemorrhagic conditions
K72	Hepatic failure
E87.2	Acidosis

^a^ Norwegian version, URL https://finnkode.ehelse.no/#icd10/0/0/0/-1

The NPR database was used to obtain data regarding hospital stay (days), outcome (hospital mortality), age and gender. Information about the total number and total duration of somatic hospital stays in Norway during the years 2011 and 2012 was also collected from the NPR, while national population data including total number of hospital deaths were retrieved from Statistics Norway. The extracted patient data were transferred to a local database (FileMaker, Inc, Pro 14.0; Santa Clara, CA, U.S.). In patients with more than five admissions during the study period, the ≥ 6.th admission(s) were excluded from analyses. In the presentation of the results, descriptive statistics for continuous variables are given as mean ± standard deviation (SD) or median and interquartile range (IQR). Annual population incidence of hospitalized sepsis was calculated as the number of patients experiencing one or more sepsis episode(s) during 2011 and 2012, divided by the sum of the total number of inhabitants in Norway during the same years. Population incidence by age and gender was compared by incidence rate ratios (IRRs) with 95% confidence intervals (CIs). Survival is illustrated by Kaplan-Meier plots and was compared between groups with log rank tests. A p-value ≤ 0.05 was considered statistically significant. The statistical analyses were performed using IBM SPSS Statistics (version 23.0; Aramonk, NY, U.S.); with the exception of the IRRs which were computed with MedCalc for Windows (version 12.7; Ostend, Belgium).

### Ethics approval

The study was approved by the Regional Committee for Medical and Health Research Ethics in Western Norway, with a waiver of informed consent (case number 2014/1922).

## Results

**Fig 2:**
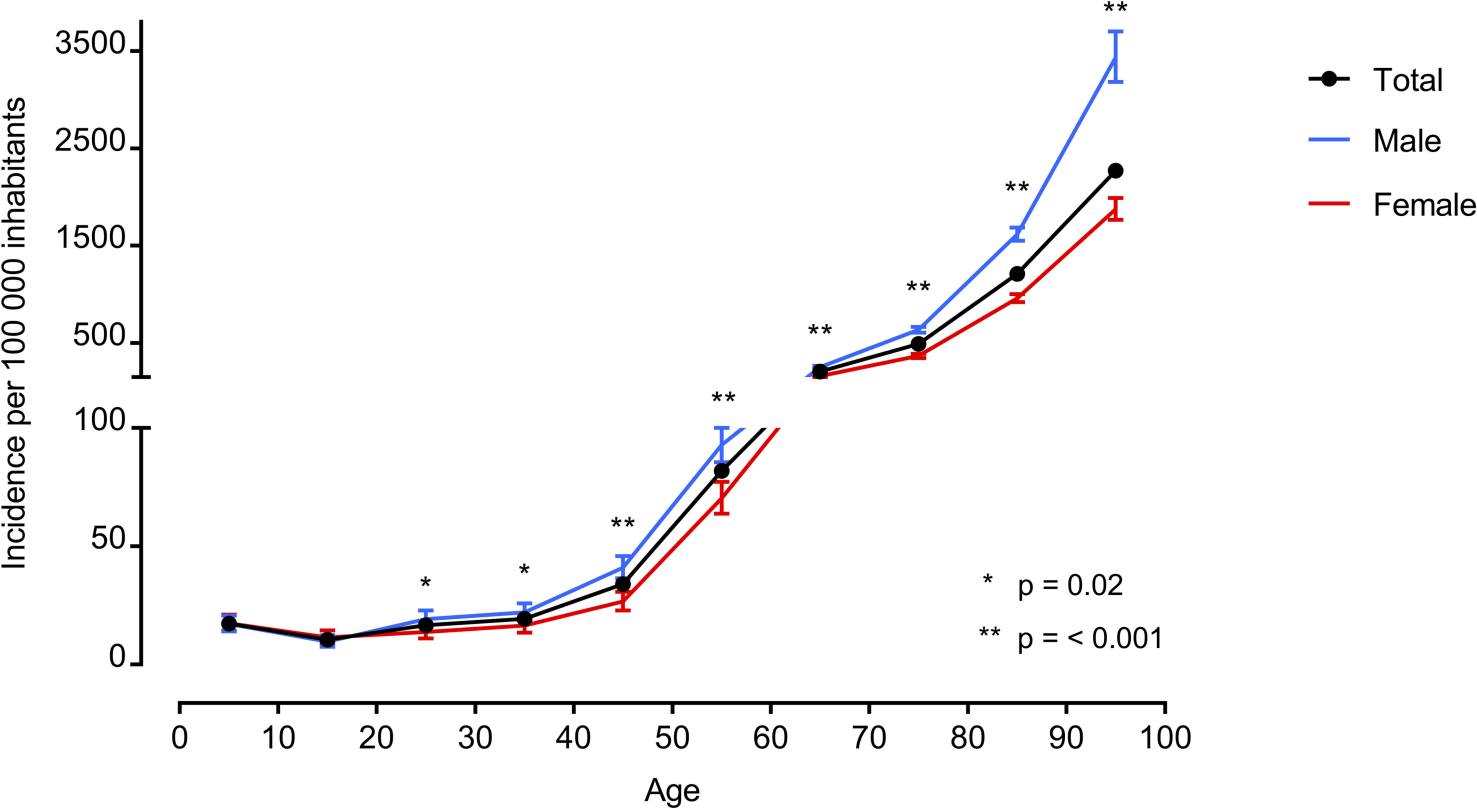
Age-specific annual incidence of sepsis hospitalizations by gender in Norway 2011–2012. Significant gender differences in incidence rate ratios were found starting from category 20–29 years and upwards, as shown in S1 Table.

During the years 2011 and 2012, we identified 18 460 sepsis admissions occurring in 13 582 individuals in Norway. Hospitalizations with sepsis constituted 1.0% of the total number of somatic hospital admissions (n = 1 767 535, Fig 1), and the annual population incidence of hospitalized sepsis was 140 per 100 000 inhabitants. The incidence showed a great age dependent increase; from 10 to 2270 patients per 100 000 inhabitants per year in different age groups (Fig 2). The increase was more pronounced among men, who reached a maximum age-specific annual incidence of 3430 per 100 000 inhabitants, while the corresponding rate for women was 1880 per 100 000. However, significant gender disparities in incidence rates were found across all adult age categories, starting from 20–29 years and upwards (S1 Table).

**Fig 3:**
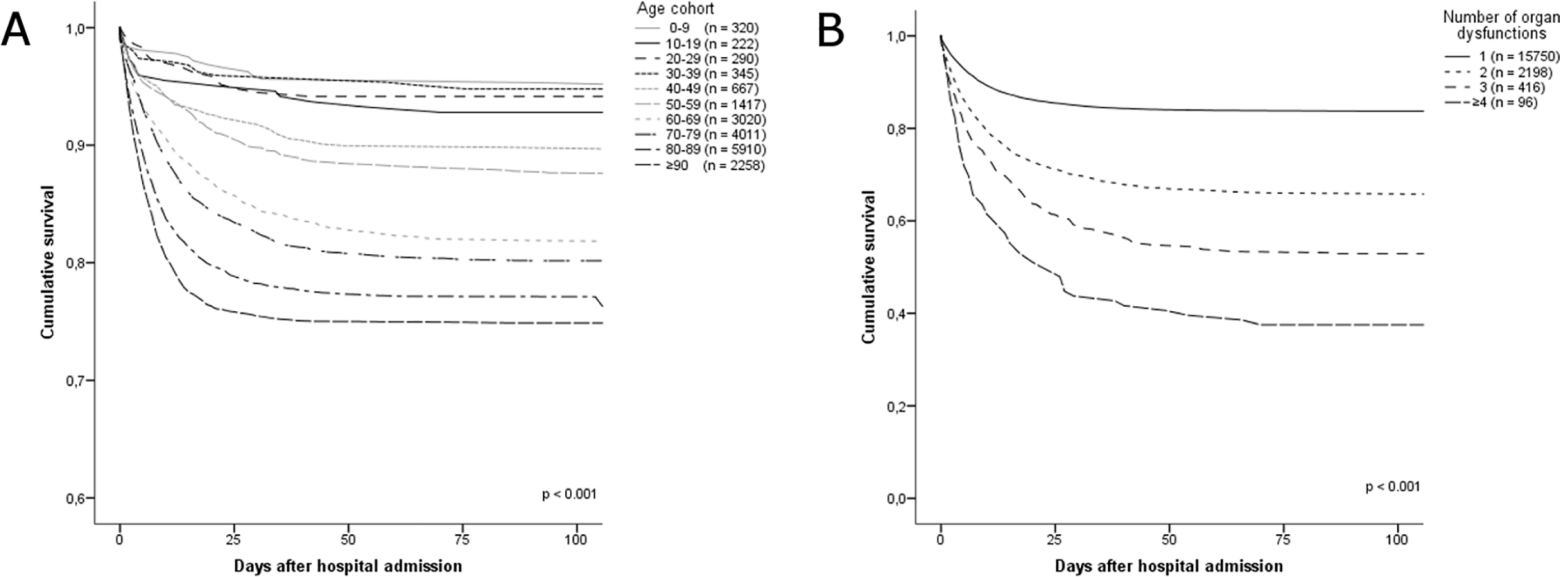
Hospital mortality for sepsis admissions in Norway 2011–2012. Kaplan-Meier plots illustrating hospital mortality for sepsis admissions in Norway during 2011 and 2012, according to A. different age cohorts and B. number of affected organ systems.

Characteristics of the study cohort are presented in Table 2. In total 82.8% of patients were ≥ 60 years and the respiratory tract was the most common site of infection. Two or more acute organ dysfunctions were documented in 14.7% of cases. The hospital mortality for sepsis admissions was 19.4%, and overall during the study period 26.4% of the included patients died while hospitalized for sepsis. Hospital mortality increased with age (Fig 3A, log rank p < 0.001) and number of organ dysfunctions (Fig 3B, log rank p < 0.001).

**Table 2:** Characteristics of patients with sepsis in Norwegian hospitals 2011–2012.

Characteristic	N (% of total) ^a^
**Gender** ^**b**^	
Male	7 327 (53.9%)
Female	6 255 (46.1%)
**Age** ^**b**^	
Median (IQR)	78 (21)
Mean ± SD	73 ± 18
**ICD-10 codes found in the primary search** ^**c**^^,^ ^**d**^	
Respiratory infections	12 932 (70.1%)
Soft tissue infections	899 (4.9%)
Genitourinary infections	822 (4.5%)
Abdominal infections	798 (4.3%)
Infection following a procedure	641 (3.5%)
Streptococcal sepsis	557 (3.0%)
Other sepsis (A41)	5 092 (27.6%)
SIRS (R65.0,1 or 9)	1 087 (5.9%)
Septic shock	735 (4.0%)
Other	159 (0.9%)
**Organ dysfunctions** ^**c**^	
Cardiovascular	8 944 (48.5%)
Respiratory	5 907 (32.0%)
Renal	4 597 (24.8%)
Hematologic	1 659 (9.0%)
Hepatic	436 (2.4%)
Metabolic	259 (1.4%)
**Number of organ dysfunctions** ^**c**^	
1	15 750 (85.3%)
2	2 198 (11.9%)
3	416 (2.3%)
≥ 4	96 (0.5%)
**Length of stay, days** ^**c**^	
Median (IQR)	9 (12)
Mean ± SD	14 ± 19
**Hospital mortality** ^**b**^	
Total	3 620 (26.4%)
Male	2 021 (27.6%)
Female	1 565 (25.0%)

^a^ if not otherwise specified.

^b^ calculated from total number of patients hospitalized with one or more sepsis episode(s) (n = 13 582).

^c^ calculated from total number of sepsis admissions (n = 18 460).

^d^ in total 23 722 primary diagnostic codes were identified; patients could have more than one code.

The total number of hospital deaths in Norway during 2011 and 2012 was 27 705, and deaths during hospital stays for sepsis constituted 12.9% of all hospital fatalities (Table 3). Furthermore, hospitalizations with sepsis accounted for 3.5% of the total admission days during the same period.

**Table 3:** Summary of total and sepsis related hospitalizations in Norway 2011–2012.

Study year	Population in Norway	Total hospital admissions	Sepsis admissions	Total patients	Sepsis patients	Sum total hospital admission days	Sum sepsis admission days	Total hospital deaths	Sepsis related deaths
**2011**	4 920 305	878 368	8 069	596 704	6 574	3 806 900	124 792	14 088	1 795
**2012**	4 985 870	889 167	10 391	601 456	7 008	3 667 016	139 679	13 617	1 791
**Sum**	9 906 175	1 767 535	18 460	1 198 160	13 582	7 473 916	264 471	27 705	3 586

If not otherwise specified, data represents number of cases (n =)

## Discussion

This nationwide retrospective register-based study from 2011 and 2012 confirms that sepsis is frequent and often fatal in Norwegian hospitals. The overall annual population incidence was 140 per 100 000 inhabitants, showed a considerable age dependent increase, and was highest among males. Sepsis admissions occupied 3.5% of the total admission days and had a mortality rate of 19.4%. The observed number of deaths corresponded to 12.9% of the total number of hospital fatalities during the study period, which to our knowledge is the first estimate of sepsis’ impact on overall hospital mortality from complete nationwide data.

The definition of sepsis was recently changed, and the term severe sepsis abandoned [7]. To facilitate the interpretation of our results, we use the word sepsis as synonymous with the new definition throughout the following discussion (i.e. life-threatening organ dysfunction caused by a dysregulated host response to infection).

This is the second nationwide retrospective study of sepsis in Norway. Compared with previous data, the most notable difference is an almost threefold increase in the annual population incidence which was estimated to 50 per 100 000 inhabitants in the year 1999 [3]. Other epidemiological studies of sepsis in Norway is restricted to a single-center, prospective study performed by the current authors in 2008 [8, 9]. Then, we detected an incidence of community acquired sepsis of 50 per 100 000 inhabitants per year. It is plausible that these observations reflect an ongoing trend of increasing sepsis occurrence, attributed to a growing number of individuals at risk for severe infection [10–13]. Register-based studies are additionally likely influenced by changes in coding patterns [14]. However, we included both codes for severe infections, SIRS, sepsis by causative microbes, and septic shock in our primary search. Thus influence of a potential shift in coding towards more frequent use of sepsis specific codes was limited.

Throughout the last two decades there have been numerous international publications on the epidemiology of sepsis. Yet, only eight countries have reported population-level incidences and only four previous studies from Europe are performed with nationwide data [1–3, 11, 15, 16]. It is well known that there are large differences in previous reports of sepsis occurrence, which partly may be explained by different study designs [1, 10, 17, 18]. Overall, our current results are in line with two recent nationwide European studies as well as a population-based study from China, while the most recent studies from the U.S. tend to report higher estimates [2, 11, 13, 15, 19–21]. This may reflect differences in health care systems as well as ICD-coding practices [17]. Also, studies from the U.S. tend report incidence as the number of sepsis *admissions* per unit of population older than18 years of age. If we use the same criteria, our corresponding rate was e.g. 270 per 100 000 population in the year 2012.

We found a slight predominance of males in our study. There was in particular a higher age-specific incidence of sepsis in males compared to females among the elderly, but significant differences in incidence rate ratios were present in all adult cohorts. Possible explanations for gender disparities in sepsis have been reviewed elsewhere [17], as similar age and gender differences in sepsis occurrence have been observed [2, 15, 22–24]. In line with studies of trends in sepsis epidemiology, our mean age of 72 years is higher than the equivalent of 58 years found in the previous nationwide report from Norway [3, 11, 23, 25]. The high average age among our patient population furthermore corresponds to recent results [11, 13, 24]. The elderly is especially predisposed to sepsis due to their high prevalence of chronic diseases, polypharmacy, repeated hospitalizations, functional loss, malnutrition, common residencies in long-term care facilities and, of course, due to age-related immunosenescence itself. Yet there is no doubt that the registered hospitalizations among the oldest patients represent cases of severe and resource demanding illness, these circumstances indicate that the elderly on average will have a greater number of diagnostic codes per hospital stay. This probably leads to a greater chance of false positive sepsis cases by use of a code-based identification strategy, and estimation of sepsis incidence is therefore especially prone to uncertainty in this subgroup of patients.

Respiratory tract infections dominated among the infectious sources of sepsis in our patients. Most of the previous register-based studies do not specify the distribution of infection codes. However, similar results were found in the U.S. in 1995, and respiratory tract infection was the most frequent infection category in recent prospective studies from both emergency department and intensive care unit settings, as well as in our previous prospective study from Norway [8, 22, 26, 27].

The number of organ dysfunctions among our patients is in the lower range compared to previous nationwide figures from Spain and the U.S. [11, 13, 20, 21]. Case inclusion in these retrospective studies was performed with fewer ICD-codes for infection and additional codes for organ dysfunction. This may have resulted in selection of more severely ill patient populations [19, 28–30]. Of interest is a Swedish study that evaluated previously used approaches for database extraction and found a lower presence of multiple organ dysfunctions among their Swedish cohort than in two reference publications from the U.S. [16, 22, 23]. Nevertheless, these findings do not reveal whether the apparent lower disease burden of sepsis in Scandinavia actually is a true reflection of the disease, or a bias from a pattern of under-coding. Previous prospective reports from Scandinavia have found a higher occurrence of multiple organ dysfunctions, but they are single-center studies from large University Hospitals [9, 26]. Prospective registration is inevitably superior in this setting, as it does not rely on compliance during discharge coding. In addition, we excluded dysfunction of the central nervous system which was present in 30–34% of the prospectively identified Scandinavian cases, due to lack of a distinct ICD-10 code.

Hospital mortality for sepsis admissions was 19.4% in our study, and 26.4% of the cohort died while hospitalized for sepsis. This is consistent with other similar recent international studies [13, 24]. Further, hospital mortality from sepsis in Norway has decreased from the previous estimation of 27.1%; despite an increase in mean age and a co-occurring decrease in the mean number of admission days [3]. The latter has also been noted elsewhere [11, 13, 20].

We found that sepsis contributed to 12.9% of the total number of hospital deaths during the study period. This is in contrast to the official cause of death statistics in Norway, where sepsis is only superficially described in the annual report based on death certificates [31]. Similarly, the corresponding report in the U.S. specifies sepsis to have caused 1.5% of all deaths in the year 2014, while a retrospective investigation of hospital mortality showed that sepsis contributed to 1 in every 2 to 3 deaths [4, 32]. Both of these U.S. estimates include patients without organ dysfunction. If we use the number of deaths found in our primary cohort (i.e. hospital fatalities among the patients with selected codes for infection, sepsis or SIRS, n = 8186), our corresponding number is 29.5%. With the exception of the mentioned retrospective report, we found no previous literature on sepsis’ influence on total hospital mortality [4]. Other researchers have used multiple causes of death data to assess the impact of sepsis on population-level all-cause mortality [33–36].This approach underestimates sepsis-related mortality compared with administrative datasets [37].

The aforementioned findings illustrate important difficulties in sepsis surveillance and reporting. Several authors have reviewed approaches for code-based identification of sepsis. Many have advised against limitation of discharge codes to diagnoses specific for sepsis and septic shock. This has been found to yield more severely ill patient populations than prospective settings, and underestimate sepsis incidence [19, 28, 30]. Furthermore, a prospective survey of sepsis in the medical emergency department at a Danish University Hospital re-identified only one in seven cases with a subsequent search based on ICD-codes [26]. The latter results are undoubtedly notable, yet the prospective inclusion is subject to some limitations such as lack of verification of sepsis beyond the ED, and an unusual distribution of organ failure (65.1% had respiratory failure, denoted as SpO2 < 92% at admission, versus only 7.4–9.2% with cardiovascular and renal failure). Just recently, a Swedish study evaluated three retrospective strategies including the previously used Norwegian method, against an intensive care unit registry [38]. In this context, one should note that all of the evaluated methods were designed prior to the introduction of specific codes for SIRS and sepsis with organ dysfunction. Although an incomplete amount of patients was identified by the Norwegian approach, it was found to be the superior strategy [3, 22, 23].

### Limitations

The main limitation of this study is its retrospective, code-based design [16, 17, 19]. In short, it encumbers our results with uncertainties due to 1) its reliance on physicians’ ability to recognize sepsis, 2) its susceptibility to under-documentation of sepsis *per se* and/or of accompanying clinical findings, and, oppositely, 3) its susceptibility to identify false positive cases because codes for organ dysfunction not necessarily originate from infection. Likewise, fatalities could be caused by another co-occurring condition. Nevertheless, our results are similar to contemporary results from a comprehensive manual review of all medical records of a Chinese population [2]. Ideally we should have used a prospective design. This is unfortunately not feasible on a national level, besides, recent data highlights that even case-based identification of sepsis may be subject to high variability [39]. We confined our search to infections of a certain severity in addition to the sepsis specific codes, and used a modest selection of acute organ dysfunctions based on the previously applied method in Norway. In light of the above discussion, we therefore consider our current criteria for inclusion reasonable.

## Conclusions

This nationwide study of sepsis in Norwegian hospitals shows an increasing occurrence compared with previous data from 1999, while hospital mortality still is considerably high. Sepsis should be recognized as an important contributor to hospital deaths, and receive attention in official reports in the future. Improvements in treatment and survival could influence population mortality. This is highly relevant, as there is reason to assume that the annual number of hospitalizations and deaths from sepsis will continue to increase due to an aging population.

## Supporting information

S1 Table Age-specific incidence rates for sepsis per 100 000 person-years at risk in Norway 2011–2012, according to gender. (DOCX)

S1 Dataset Hospitalization data obtained from the Norwegian Patient Registry (published with permission). (XLSX)

## Abbreviations

NPR: Norwegian Patient Registry
ICD-10: International Classification of Diseases 10^th^ revision
U.S.: United States
SD: standard deviation
IQR: interquartile range
IRR: incidence rate ratio
CI: confidence interval
SIRS: systemic inflammatory response syndrome
