# Supplementary material for: Epidemiology and impact on all-cause mortality of sepsis in Norwegian hospitals: A national retrospective study
Source: PLoS One. 2017 Nov 17;12(11):e0187990. doi: 10.1371/journal.pone.0187990 (PMC5693291; doi:10.1371/journal.pone.0187990)
Supplement: S1 Table — (DOCX) [file pone.0187990.s001.docx]

**S1 Table. Age-specific incidence rates for sepsis per 100 000 person-years at risk in Norway 2011 - 2012, according to gender**

| **Age cohort** | **Incidence rate, male** | **Incidence rate, female** | **Incidence rate ratio (95% CI)** | **p-value** |
| --- | --- | --- | --- | --- |
| 0-9 | 17 | 17 | 0.98 (0.74-1.30) | p = 0.89 |
| 10-19 | 10 | 11 | 0.85 (0.60-1.21) | p = 0.35 |
| 20-29 | 19 | 14 | 1.40 (1.06-1.86) | p = 0.02 |
| 30-39 | 22 | 17 | 1.34 (1.04-1.73) | p = 0.02 |
| 40-49 | 41 | 27 | 1.54 (1.28-1.86) | p < 0.001 |
| 50-59 | 93 | 70 | 1.32 (1.17-1.50) | p < 0.001 |
| 60-69 | 252 | 159 | 1.59 (1.45-1.73) | p < 0.001 |
| 70-79 | 635 | 366 | 1.73 (1.61-1.87) | p < 0.001 |
| 80-89 | 1618 | 959 | 1.69 (1.59-1.79) | p < 0.001 |
| 90 + | 3434 | 1876 | 1.83 (1.66-2.02) | p < 0.001 |
| **Total** | 148 | 126 | 1.17 (1.13-1.21) | p < 0.001 |
